# Leukemic Transdifferentiation: From Pathological Plasticity to Dendritic Cell-Based Immunotherapy

**DOI:** 10.3390/biomedicines13123099

**Published:** 2025-12-16

**Authors:** Joanna Dubis, Aleksander Czogalla, Kazimierz Kuliczkowski, Aleksander F. Sikorski

**Affiliations:** 1 Research and Development Centre, Regional Specialist Hospital at Wrocław, Kamieńskiego 73a, 51-124 Wrocław, Poland; joanna.dubis@wssk.wroc.pl (J.D.); kazkul@wp.pl (K.K.); 2 Department of Cytobiochemistry, Faculty of Biotechnology, University of Wrocław, Joliot-Curie 14a, 50-368 Wrocław, Poland; aleksander.czogalla@uwr.edu.pl

**Keywords:** transdifferentiation, leukemia, dendritic cells, partial transdifferentiation, leukemic cell plasticity, immunotherapy

## Abstract

Transdifferentiation, also known as direct reprogramming, is the transformation of one terminally differentiated cell type into another mature cell type, while bypassing the stage of pluripotency. In leukemia, this phenomenon has a dual significance: on the one hand, it is an adaptive mechanism driving tumor survival and resistance to treatment, and on the other, it offers a potential opportunity for innovative therapies. Of particular interest is the directional transdifferentiation (mostly partial) toward dendritic cell-like phenotypes, which increases the immunogenicity of cancer cells. Mastering this process could define a new generation of immunotherapies that leverage the inherent plasticity of leukemic cells to achieve therapeutic benefits. In this brief review, we attempt to gather information concerning the molecular mechanism of this process and point to the role of dendritic cells as a crucial element of anticancer, particularly anti-leukemia innate and acquired, immunity. Thus, in vitro and in vivo techniques of inducing transformation of the leukemia cells into cancer antigen-presenting cells and the application of these technologies in current and future therapies are discussed.

## 1. Introduction

Leukemias are proliferative diseases of the hematopoietic system and are classified into acute and chronic types. Acute leukemias involve the proliferation of progenitor cells, while chronic leukemia cells resemble mature lymphocytes or myeloid cells, but their intrinsic defects cause them to divide excessively. The most common acute leukemias include lymphoblastic and myeloblastic leukemias, while chronic leukemias include chronic myeloid and lymphocytic leukemias. Treatment for acute leukemias consists of the following stages: remission induction, maintenance therapy, relapse treatment, and bone marrow transplantation. Induction therapies that influence survival are listed below.

Acute lymphoblastic leukemias (ALL, B or T lymphocytic) involve the proliferation of B or T lymphocyte progenitor cells (lymphoblasts). In addition to cytogenetic changes such as chromosome number or translocations, i.e., t(12;21), t(1;19), and t(9;22), genetic changes such as *BCR::ABL1*-like, *IKZF1*, *PAX5*, *CDKN2A/B*, *EBF1*, *RB1*, *TP53*, JAK/STAT pathway (*JAK1/2*, *IL7R*, *CRLF2*), and RAS/RTK pathway (*FLT3*, *KRAS*, *NRAS*) gene mutations and *KMT2A*– rearrangement often underlay the disease. Cytogenetic and molecular changes are classified into standard risk and poor risk [1]. Treatment of B lymphocytic ALL is based on existing drugs and the presence or absence of the Philadelphia chromosome (Ph+, i.e., *BCR::ABL*-like). In the presence of a Ph+ chromosome, a specific tyrosine kinase inhibitor is added to cytostatic therapy. As the disease progresses, a bivalent anti-CD3 and anti-CD19 antibody is added as an immunotherapeutic. Survival time depends on age and other factors. Younger individuals (15–39) have a 5-year survival rate of 85%, and the survival time decreases with age [2,3].

Acute myeloblastic leukemia involves the proliferation of myeloid progenitor cells. Cytogenetic changes mostly include the following: inv 16, inv 3, (16;16), t(8;21), t(15;17), t(9;11), t(3;3), t(6;9), t(9;22), t(8;16), trisomy 8, and complex changes in chromosome 3 monosomy 5, 5q-, 7, 7q-, and 11q23. Observed genetic abnormalities include no changes or mutations in the following genes: *NPM1, FLT3-ITD*, *FLT3-TKD*, *CEBPA*, *TP53*, *IDH-1*, *RUNX1*, *ASXL1*, *BCOR*, *EZH2*, *SF3B1*, *SRSF2*, *STAG2*, *U2AF1*, and *ZRSR2*. The National Comprehensive Cancer (NCCN) rates these lesions according to risk as favorable, intermediate, and weak/unfavorable. Treatment is determined depending on the risk group, but in general, it includes cytostatic therapy, the use of anti-CD33 antibodies, and, if *FLT3* or *IDH-1* mutations are present, specific inhibitors of these enzymes. Hypomethylating agents and the Bcl-2 blocker venetoclax are used. These treatments allow for a 5-year survival rate ranging from 1% in individuals over 80 years old to 60% in individuals under the age of 40 years [4].

Myeloblastic leukemias include acute promyelocytic leukemia and a rare neoplasm of blastic plasmacytoid cells [5]. Acute promyelocytic leukemia is caused by a 15;17 translocation, which fuses the gene for RAR receptor on chromosome 17 with the *PML* gene. This makes the cells less sensitive to retinoids, leading to promyelocyte proliferation [6]. Treatment includes retinoids, which break the block, and arsenic trioxide, which stimulates apoptosis in, among other cells, promyelocytes with t(15;17). Four-year survival can reach 91% [7].

Chronic myeloid leukemia is caused by a translocation of part of the chromosome 22 gene (break cluster region) that fuses with the *ABL* gene on chromosome 9, creating the p210 or p185 protein. The ABL protein is a tyrosine kinase, and the attachment of a portion of chromosome 22 activates it, stimulating myeloid cell proliferation, resulting in the appearance of increased number of cells with normal-like morphology in the smear. Tyrosine kinase inhibitors are used in treatment, the first of which was imatinib, followed by several generations of these drugs. The 8-year survival rate is over 60% [8].

Chronic lymphocytic leukemia/small-cell lymphoma and chronic myeloid leukemia-like are characterized by the excessive proliferation of morphologically normal lymphocytes, but this increase is caused by the changes at the cytogenetic and molecular levels [9]. Cytogenetic changes include the absence of cytogenetic changes, del(13q), trisomy 12, del(11q), and del(17p). Molecular abnormalities include mutations in variable Ig heavy chain, *TP53* with/without del(17p), and mutations in *ATM*, *BIRC3*, *NOTCH1*, *SF3B1*, *BTK*, *CARD11*, *PLCG2*, and *BCL2* genes. At diagnosis, the disease is classified into five stages (0 to IV) according to the Rai system or into three stages (A, B, and C) according to the Binet system [10,11]. Lymphoma is classified along with chronic lymphocytic leukemia because the treatment and management are similar. Lymphoma staging is determined by the modified Ann Arbor classification [1,3]. Treatment is indicated for clinically active disease according to its stages. Initial treatment depends on the presence of del(17p)/*TP53* mutation. Generally, a Bruton’s kinase inhibitor, e.g., acalabrutinib with an anti-CD20 antibody (obinutuzumab), or venetoclax with an anti-CD20 antibody, or a combination of venetoclax, acalabrutinib, and obinutuzumab, is used [1]. The 5-year survival rate is 90% for patients under 69 years of age, and 80% for those aged 70 and older [3].

The immune response against leukemias differs fundamentally from the typical anticancer response observed in solid tumors. These differences stem from the nature of the transformation, the location of the disease process, and the microenvironment in which the tumor cells interact with the immune system. In leukemias, hematopoietic cells undergo neoplastic transformation, which physiologically function as effectors of antitumor immunity. The immune system is therefore forced to recognize and eliminate its own immune cells which have lost proliferative control and acquired leukemic characteristics. This phenomenon leads to the development of immune tolerance and the weakening of immune surveillance mechanisms [12].

Unlike solid tumors, leukemias do not form organized tumor structures but develop diffusely within the bone marrow and blood. The process of immune recognition thus takes place in an environment with strong immunoregulatory properties, which favors the maintenance of hematopoietic homeostasis but at the same time limits the effectiveness of the antitumor response [13].

One of the hallmarks of leukemia is the impairment of immune surveillance mechanisms. Leukemic cells demonstrate the ability to actively suppress the immune response by expressing suppressor molecules such as PD-L1, GAL-9, and CD47, and secreting immunosuppressive cytokines, including TGF-β and IL-10. Furthermore, they induce the development of regulatory T cells (Tregs) and myeloid suppressor cells (MDSCs), which limit the effector activity of T and NK lymphocytes. As a result, the bone marrow and blood microenvironment become immunosuppressive, favoring tumor cell survival [14].

Antitumor response in leukemias is also hampered by the low immunogenicity of tumor antigens, which are often derived from proteins naturally occurring in hematopoietic cells, such as WT1, PR1, or RHAMM. The ability of T lymphocytes to recognize these antigens depends on their presentation in the MHC class I complex, which is sometimes impaired in leukemic cells [15].

In this review, we aim to present the current state of knowledge about transdifferentiation, both as one of the mechanisms of pathological plasticity in leukemias (lineage infidelity/lineage switch) and as a potential therapeutic tool. Particular attention is paid to directional transdifferentiation toward dendritic cell (DC)-like phenotypes, which increase the immunogenicity of cancer cells. Moreover, we discuss the potential for integrating these strategies with existing immunotherapies, while taking into account their efficacy and safety.

## 2. Maintenance of the Lineage Identity of Leukemic Cells

Leukemic cells exhibit a high level of heterogeneity and phenotypic plasticity, which increases their adaptive capacity and resistance to treatment [16,17]. Maintaining cellular identity requires constant epigenetic control. Mutations in genes regulating chromatin modifications and DNA methylation, such as *TET2*, *DNMT3A*, *IDH1/2*, and *EZH2*, are common in leukemias. These mutations disrupt the balance between active and repressive states of the epigenome. This leads to a loss of lineage stability and promotes spontaneous transition into alternative phenotypes [18,19,20]. Metabolic reprogramming is also observed, with leukemia cells switching from oxidative phosphorylation to aerobic glycolysis and pentose phosphate pathways. These changes provide precursors for the biosynthesis of nucleotides, proteins, and lipids and influence epigenetic modifications by regulating the levels of metabolites such as α-ketoglutarate, NAD^+^, and acetyl-CoA (for current reviews see, e.g., [21,22,23,24,25]). This coupled epigenetic–metabolic plasticity creates an environment conducive to maintaining hybrid states in leukemic cells and a loss of lineage identity.

In the context of adaptation to environmental and therapeutic pressures, metabolic reprogramming emerges as a key regulator of cellular identity through its direct impact on the epigenetic landscape. The shift from glycolysis to oxidative phosphorylation (OXPHOS) results in the altered availability of key metabolites, including α-ketoglutarate and NAD^+^, which modulate the activity of epigenetic enzymes. Increased levels of α-ketoglutarate promote the activity of TET family DNA demethylases, including TET2, while changes in methyl metabolism affect the DNMT3A function, leading to the remodeling of DNA methylation patterns and chromatin organization [26,27]. In leukemic cells. OXPHOS-related metabolic reprogramming is often induced by therapeutic pressure and characterizes subpopulations of leukemia stem cells and treatment-resistant cells that exhibit increased metabolic flexibility and capacity for long-term survival [28]. A similar metabolic profile is promoted by the bone marrow microenvironment and cellular quiescence, contributing to the destabilization of cell lineage memory and increased cell fate plasticity, thus enabling transcriptional reconfiguration, including transdifferentiation processes [29,30,31].

The cells of the hematopoietic system, unlike other tissues, are characterized by an extraordinary ability to change their identity via several expression profile pathways. Among these, phenomena such as dedifferentiation, transdifferentiation, and lineage switching are particularly important, reflecting varying degrees of loss or modification of the differentiation program. Dedifferentiation involves regressing a cell to a less differentiated, progenitor, or “stem-like” state, accompanied by the loss of expression of terminal differentiation genes and the reactivation of developmental pathways such as Notch, Wnt, or Hedgehog [32,33,34].

Lineage switching, defined as the “immunophenotypic transformation of cell clones”, represents a change in the dominant differentiation program within hematopoiesis, often observed in the context of leukemias, e.g., from ALL to AML (most cases) and from AML to ALL (rather rare) [35]. One of the suggested molecular mechanisms is therapy-induced microevolution, i.e., a latent clone that survived therapy can be selected for expansion, while the dominant, mutated blasts are eradicated, or the dominant clone may acquire additional mutations to evolve into another, possibly pathological, one. There are numerous cases in the literature, e.g., [36,37], which show that CD19 CAR-T therapy accelerated the existing myeloid lineage evolution via ANXA1-FPR1/2 axis expression from HSCs with 6q aberrations.

On the other hand, the microenvironment (mainly bone marrow) created by several cell types regulates the maintenance and development of hematological progenitors and can also trigger pathological changes in the cells. The current data indicate decreased production of HSC-promoting factors such as CXCL12, SCF, and ANGPT1 by stromal cells, which harms healthy HSCs and favors leukemic blasts. Pro-inflammatory signals such as TNFα, IL-1, and IL-6 are also known to drive myeloid malignancies and bone marrow fibrosis [38]. For example, Wang et al. demonstrated that a loss of Notch signaling increases the expression of miR-155, which directly acts on the NF-κB inhibitor and activates NF-κB resulting in the increased production of pro-inflammatory cytokines including G-CSF and TNFα in endothelial bone marrow cells. This, in turn, directly drives a myeloproliferative neoplasm-like disease [39]. It is worth mentioning that chronic inflammation is considered as one of the leukemogenic factors, as it causes DNA damage in hematopoietic stem and progenitor cells, thus contributing to the formation of preleukemic clones [40,41].

Taken together, the available evidence suggests that cell lineage switching in leukemias may result from both Darwinian selection of pre-existing clones under therapeutic pressure and active, microenvironment-driven cellular plasticity associated with chronic inflammation and the disruption of the bone marrow niche [16,31]. It is increasingly recognized that these mechanisms operate in concert, leading to dynamic changes in leukemic cell identity during disease progression and treatment.

## 3. Transdifferentiation

### 3.1. The Event

Transdifferentiation represents a direct transformation between two mature cell types, e.g., from the lymphoid to the myeloid lineage, and is the result of a switch in transcription factor (TF) networks (e.g., PAX5 ↔ C/EBPα) and the restructuring of the epigenome [31,42]. According to the definition proposed by Jopling et al. [42], classical transdifferentiation refers to the direct reprogramming of one terminally differentiated cell type into another, possibly mature, cell type, bypassing the stage of pluripotency. It leads to the formation of a new, stable cellular identity, fixed at both the transcriptional and epigenetic levels [43,44,45]. This process leads to a profound remodeling of the gene program and a complete change in the cellular phenotype.

Classic examples of this phenomenon include the conversion of fibroblasts into cardiomyocytes [46], the transformation of fibroblasts into neurons [47], and the gradual induction of B lymphocyte transition into macrophages [48]. As a rule, these phenomena are exceptional in healthy mammalian tissues and require artificial induction via the expression of specific TFs, such as C/EBPα, GATA4, MEF2C, TBX5, or ASCL1, which initiate chromatin opening, the extinction of the existing transcriptional program, and the activation of a new set of genes.

Classical transdifferentiation involves complete transcriptional and epigenetic reprogramming, leading to the stable establishment of a new phenotype. At the molecular level, this process extends over several stages. During the initiation phase (1), pioneer TFs (e.g., C/EBPα, GATA4, FOXA1, PU.1, SOX2) bind to closed chromatin and open new regulatory regions, simultaneously extinguishing the existing gene expression program [49,50,51]. The second (2) is a transition phase in which the features of the initial and target phenotype coexist, and the epigenome undergoes profound reorganization, including the DNA and histone modifications, nucleosome reorganization, and remodeling of the spatial structure of chromatin. Regulatory regions associated with the new genetic program acquire activating histone modifications, such as H3K27ac and H3K4me1/3, while the earlier program is repressed by H3K27me3 (trimethylation of lysine 27 on the histone H3 protein) [50,52,53]. The final (3) phase is the stabilization, leading to the consolidation of the new transcriptional program and the loss of the original identity [49,50,53]. This occurs through the formation of positive feedback loops in TF networks and the reorganization of interactions between enhancers and promoters. As a result, expression of the target program becomes self-sustaining, and the cell gains phenotypic stability and resistance to reversion to the initial state [52,53,54]. This phase distinguishes full transdifferentiation from states of transient plasticity, among them partial and spontaneous transdifferentiation, as it guarantees the durability and functionality of the new cellular identity.

All stages of transdifferentiation are tightly modulated by the tissue microenvironment. Cytokines such as TGF-β, IL-6, and TNF-α, growth factors including EGF and FGF, extracellular matrix components, and the mechanical stimuli influence the activity of epigenetic enzymes, cytoskeletal organization, and nuclear signaling [55,56]. Switching between glycolysis and oxidative phosphorylation also leads to changes in the levels of key metabolites such as S-adenosylmethionine, α-ketoglutarate, NAD^+^, and acetyl-CoA. These compounds act as essential substrates and cofactors for enzymes responsible for DNA methylation and demethylation and histone modifications [27]. As a result, a new, stable epigenetic–transcriptional architecture is created perpetuating an alternative cellular identity [56,57].

Classical transdifferentiation is primarily an experimental model that demonstrates that cell identity is not an irreversible state, although in normal mammalian tissues and organs, this process occurs quite rarely under natural conditions and is limited to exceptional regenerative or pathological situations in which a complete change in the cell’s transcriptional program occurs.

The literature in this field, however, indicates that the term “transdifferentiation” lacks a clear definition. Initially, it referred exclusively to the aforementioned classical conversion between fully differentiated cells, but now the concept also encompasses partial and reversible processes observed under physiological conditions, such as hematopoiesis, tissue regeneration, and cancer cell adaptation, to name a few [31,52,58,59,60]. Therefore, the literature divides transdifferentiation into physiological (natural), partial, and classical, with the boundaries between these categories being blurred and often dependent on the biological context [61,62]. Moreover, this term is sometimes used interchangeably with the concepts of lineage plasticity, lineage conversion, or lineage switching, especially in the research on hematopoiesis and cancer [63].

The lack of clarity in nomenclature reflects the complexity of this phenomenon and poses a significant challenge to the interpretation of research results. Therefore, the need to standardize terminology and distinguish between transient states of cellular plasticity and fully functional transdifferentiation is increasingly emphasized [64,65].

### 3.2. Evolutionary Conservation

In invertebrate and lower vertebrate organisms, transdifferentiation is a natural regenerative mechanism that is an integral part of tissue homeostasis, which occurs spontaneously and allows for the regeneration of lost structures by directly transforming mature cells into other functional types [66,67,68,69,70,71,72,73]. It relies on the conserved genes controlling cell fate, such as *GATA*, *C/EBPα*, *PU.1*, *RUNX1*, *SOX*, *FOXA*, *PAX*, and regulators of the Wnt, Notch, TGF-β, and FGF pathways [74,75,76]. While, in simpler organisms, these networks remain active, in mammals, they have been partially silenced by evolution toward cell stability and specialization. In mammals, transdifferentiation is rare and requires artificial induction by TFs such as C/EBPα, GATA4, MEF2C, or TBX5, occurring physiologically only in response to stress or tissue damage [46,47,77]. A few cases of natural transdifferentiation have been described in the liver, pancreas, and intestinal epithelium, where cell conversion occurs within a single tissue [73,78,79].

In hematopoiesis, particularly lymphoid and myeloid, as well as in cell lineage differentiation, which is important for the development of leukemia and cell lineage switching/transdifferentiation, TFs include RUNX1, C\EBPα, and PU.1. Only RUNX1 (CBFα, AML-1), encoded by the gene of the same name, which is a subunit of the CBFα/β heterodimer involved in the development of blood stem cells (HSCs) (in the absence of this protein, hematopoietic stem cells are not formed) [80], is highly conserved in evolution. Its ortholog was discovered even in *Nematostella,* meaning that this gene and its isoforms are present in all *Metazoa* and function as a dimeric TF with CBFβ, which was also detected in the studied organism [81]. The remaining two TFs evolved later, as their orthologs can be found in vertebrates. Briefly, PU.1 (SP1), a TFand the master regulator of hematopoiesis, encoded by the *SPI1* gene, activates gene expression during myeloid and B lymphoid cell development. It binds to the PU-box, a purine-rich sequence located near the promoters of target genes, and together with other TFs and cofactors regulates their expression (reviewed by [82]). It may also regulate alternative splicing of target genes. This gene is conserved from chondrichthyans to mammals, and a gene with homology limited to the DNA-binding domain has been detected in the lamprey (a jawless vertebrate, *Petromyzon marinus*) [83].

C/EBPα, encoded by the intronless *CEBPA* gene, is a TFcontaining a basic leucine zipper (bZIP) domain that binds the CCAAT motif in target gene promoters. This protein forms functional homodimers as well as heterodimers with CCAAT/EBPβ and γ. In hematopoiesis, this protein plays a key role in the activation of early myeloid gene expression and in the repression of nonmyeloid genes [84]. The available experimental data indicate that artificially activated C/EBPα, in cooperation with C/EBPβ or PU.1, converts B or T cell progenitors into myeloid ones capable of differentiating into monocytes/granulocytes in vitro [85]. Moreover, C/EBPα/β in the presence of PU.1 is able to transdifferentiate fibroblasts into myeloid progenitors, whereas in the presence of PPARγ and SREBP-1 into adipocytes [86,87]. The *CEBPA* gene/C/EBPα protein appears to be conserved in vertebrates and evolved even later than PU.1, as only a bZIP domain-containing c/ebp family member with myeloid specificity from *Danio rerio* has been cloned [88]. Thus, transdifferentiation in the mammalian system, particularly the hematopoietic system, may be driven by both metazoan-conserved and vertebrate-specific pathways.

## 4. Transdifferentiation Is One of the Events Responsible for the Plasticity of Leukemic Precursors

Partial, spontaneous transdifferentiation in hematopoiesis and leukemias plays a dual biological role. It supports the mechanisms of regulation and adaptation of the immune response, while on the other hand, it can promote pathological phenomena, such as leukemic cell escape from immunotherapy.

### 4.1. Partial, Spontaneous Transdifferentiation in Hematopoiesis

In hematopoiesis, this is a process in which hematopoietic cells change their transcriptional program and phenotype without fully losing their original cell lineage identity. Unlike classical transdifferentiation, complete transition does not occur, i.e., cells adopt characteristics of an alternative type while retaining elements of the original program. This results in a hybrid state permitting cells to exhibit increased plasticity and adaptability to microenvironmental signals [31,52,89,90,91,92,93]. In the literature, this term is sometimes used interchangeably with lineage plasticity or lineage conversion, but its key feature is a partial and reversible switch in the differentiation program that does not require full cellular reprogramming [31,48,58,94].

Under physiological conditions, partial, spontaneous transdifferentiation does not occur randomly but is an adaptive mechanism activated in situations of hematopoietic stress, such as infection, blood loss, or recovery from chemotherapy [13]. This process is reversible and tightly controlled by the changes in the activity of a network of TFs (PU.1, C/EBPα, GATA2, RUNX1, and IKZF1), which are followed by chromatin reorganization. Subtle changes in the balance of activity of these factors may be sufficient to switch the direction of differentiation, for example, between the monocytic and dendritic lineages [89]. The main signaling pathways, Notch, Wnt, MAPK, and TGF-β, are also involved in this process, integrating microenvironmental signals from the bone marrow, such as SCF, IL-6, TNF-α, and CXCL12, with the activity of TFs. It is therefore tightly regulated, and physiological plasticity distinguishes it from the pathological transdifferentiation observed in leukemias.

### 4.2. Spontaneous Transdifferentiation in Leukemia

In cancer, spontaneous transdifferentiation loses its physiological, controlled nature and becomes an adaptive mechanism that drives the heterogeneity and resistance of cancer cells. In hematopoietic malignancies, cells can switch differentiation programs between hematopoietic lineages, for example, from lymphoid to myeloid, which clinically manifests as a lineage switch [95,96,97,98,99,100,101]. Partial, spontaneous transdifferentiation, best described in leukemias, has also been observed and documented in solid tumors by several teams (e.g., [102,103,104], reviewed in [105])**.**

In leukemias, this phenomenon results in hybrid states characterized by the co-activation of competing programs, termed lineage infidelity [52,106]. Mutations in genes *RUNX1*, *TET2*, *DNMT3A*, *IDH1/2*, *ASXL1*, and *EZH2* additionally destabilize the epigenome, weakening the so-called “lineage memory” and lowering the threshold for phenotype switching [18,107]. Support from the bone marrow microenvironment, including IL-6, TNF-α, CXCL12 signals, and the activation of the Notch pathway, amplifies transitional states and stabilizes intermediate phenotypes, while promoting metabolic resistance [3]. As a result, epigenetically locked plasticity is perpetuated, which, instead of serving regeneration, becomes a trigger for progression, drug resistance, and relapse [108,109].

The clinical significance of this process is obvious. The plasticity of leukemic cells favors the selection of resistant variants and allows for a change in lineage program, both in the course of the disease and under the pressure of treatment. Both lineage infidelity, i.e., the co-expression of markers characteristic of different lineages, and full lineage switch, a pathological transdifferentiation that perpetuates the new phenotype and paves the way for progression and relapse, are observed. This phenomenon is driven by the disruption of the TF network (weakening of B-lineage gatekeepers such as PAX5, EBF1, or E2A, with the simultaneous enhancement of the PU.1/C-EBP/IRF axis), epigenetic instability resulting from *DNMT3A*, *TET2*, *IDH1/2*, *ASXL1*, and *EZH2* mutations, and myeloid niche signals (CXCL12–CXCR4, IL-6, TNF-α, and Notch), which altogether lower the switching threshold and promote the consolidation of intermediate states [110,111].

One of the best documented phenomenon is the conversion of B-ALL (B-acute lymphoblastic leukemia) to a myeloid phenotype or MPAL (Mixed Phenotype Acute Leukemia) after anti-CD19 immunotherapy, where recurrent lineage switch after CAR-T or bispecific antibody (BiTEs) therapy is associated with the loss of CD19 and appearance of myeloid features, which correlates with the attenuation of the PAX5 axis and the amplification of the PU.1/C-EBP axis [20,112,113,114,115,116]. Another example is a B cell tumor, which can undergo histiocytic or dendritic transformation, as evidenced by the conversion of DLBCL (Diffuse Large B Cell Lymphoma), FL (Follicular Lymphoma), or B-ALL into histiocytic sarcoma or dendritic cell sarcoma [98,117,118,119]. In acute myeloid leukemias, the phenomenon of “dendritization,”, i.e., the presence of plasmacytoid (pDC)-like DCs within AML, has demonstrated the possibility of generating ex vivo dendritic cells of leukemic origin, which indicates a trend toward the antigen presentation program [120,121]. This is complemented by observations within the histiocytic–dendritic spectrum, including LCH (Langerhans cell histiocytosis) transformation into Langerhans cell sarcoma or the demonstration of clonal relationships between different types of dendritic and histiocytic neoplasms, which emphasizes the existence of a real conversion in the monocyte–DC–macrophage axis [98,122,123]. Finally, proof-of-principle evidence shows that the forced expression of *C/EBPα* together with *PU.1* can convert mature B cells into macrophages or dendritic cells, indicating a mechanistic vulnerability of hematopoietic lineages to reprogramming [48,124,125]. The practical implications of this phenomenon are of fundamental clinical importance. Pathological transdifferentiation masks therapeutic targets, for example, through the loss of CD19 or CD22 epitopes, alters cell sensitivity to treatment by switching to the myeloid program or MPAL, increases tumor heterogeneity, and complicates monitoring of minimal residual disease (MRD). As a result, it reduces the durability of remission, even if the initial response to treatment is good. Therefore, translational strategies are gaining increasing importance. These include early detection of switching signals in MRD analysis, enhancing lineage guards and epigenetic control to stabilize cellular identity, and targeted reprogramming toward highly immunogenic states. This approach offers the opportunity to leverage the plasticity of leukemic cells to “reprogram” them to increase their effectiveness.

Transdifferentiation appears to have a dual significance. On the one hand, it promotes cancer progression and resistance; on the other hand, it may constitute a potential therapeutic target if this process is directed toward enhancing antigen presentation and restoring immunological control. The latter should be understood as the ability of the immune system to effectively recognize cancer cells, eliminate them, and maintain long-term surveillance over them.

Transdifferentiation in the hematopoietic system is a subject to specific directionality resulting from the architecture of regulatory networks and the epigenetic structure of blood cells [52,93]. The monocyte–DC–macrophage pathway remains the most susceptible to changes. In this system, an appropriate set of TFs (PU.1, C/EBP, IRF), high chromatin accessibility, and susceptibility to innate signals and cytokines (GM-CSF, M-CSF, IL-4, IL-13, IL-6, TNF-α) enable rapid and partially reversible phenotypic switches that occur without the need for dedifferentiation [126,127,128]. Among lineage conversions, the lymphoid-to-myeloid transition occurs more frequently and readily, particularly in the case of B cell transformation into macrophages or dendritic cells. This phenomenon results from asymmetry in regulatory barriers, as it is relatively simple to weaken B cell identity gatekeepers, such as PAX5 and EBF1, and shift the balance toward the PU.1/C-EBP axis. The reverse process, however, requires the re-establishment of the lymphopoietic program, including the activation of PAX5, EBF1, and E2A, expression of *RAG1/2* genes, and remodeling of Ig/TCR loci [52,129]. This phenomenon is supported by both experimental data and clinical observations. Laboratory studies have shown that the forced expression of *C/EBPα* and *PU.1* leads to the conversion of B cells into macrophages [130,131,132,133].

In clinical practice, the transformation of B cell tumors into a histiocytic or dendritic phenotype is observed, as well as a more frequent lineage switch in ALL toward a myeloid or MPAL phenotype [20,48,112,117,118,125,134].

## 5. Dendritic Cell Subtypes and Their Role in the Anti-Leukemic Response

DCs constitute a diverse population of specialized antigen-presenting cells (APCs) that bridge innate and adaptive immunity. In the context of leukemia, they play a key role in the initiation, modulation, and maintenance of the immune response. Their activity and impact on the course of the disease are determined by the properties of individual subtypes and the bone marrow microenvironment in which DCs interact with leukemic cells [127,135]. Two major classes of dendritic cells can be distinguished, i.e., plasmacytoid pDCs and conventional cDCs. Four DC precursors, CD34 and hematopoietin receptor-positive, were found, i.e., (1) GDMP (granulocyte, monocyte, and DC progenitor) cells, (2) MDP (monocyte and DC progenitor) cells, (3) CDP (common DC progenitor) cells, and (4) pre-DC (DC precursor) cells [136,137,138,139,140].

pDCs are a major source of type I interferons (IFN-I), which enhance the activity of CD8^+^ T cells and NK cells [141]. In the leukemic microenvironment, pDCs often undergo functional silencing and adopt a tolerogenic phenotype, which promotes immunosuppression and disease progression [142]. Restoring their ability to produce IFN-I may increase tumor immunogenicity and enhance the efficacy of immunomodulatory therapies, especially when combined with STING and TLR agonists [141,143].

Conventional DCs include primarily cDC1, cDC2, and DC3s. Of all DC subtypes, conventional type 1 dendritic cells (cDC1s) are particularly important, specializing in the cross-presentation and activation of cytotoxic CD8^+^ T lymphocytes [126,144,145]. These cells recognize and phagocytize dead leukemic cells, apoptotic bodies, and antigens released as a result of cytotoxic therapies such as chemotherapy or radiotherapy [146]. This process is possible due to the presence of receptors recognizing danger signals (DAMPs) and the so-called “eat-me” signals, including the exposure of phosphatidylserine on the cell surface [147,148]. cDC1s are characterized by low endosomal acidification, the increased production of reactive oxygen species (ROS), and the ability to transfer antigens from endosomes to the cytosol, where they undergo proteasomal processing and are then presented in a complex with MHC class I molecules. These properties constitute the basis for DC1s’ unique ability to cross-present antigens, a process in which extracellular antigens derived from dead or apoptotic leukemic cells are presented in the context of MHC class I molecules. This allows the activation of naive CD8^+^ T cells, which otherwise respond only to endogenous antigens. CD8^+^ T cells activated by DCs recognize MHC class I–antigen complexes and eliminate leukemic cells through cytotoxic mechanisms mainly based on perforin and granzyme. This makes cross-presentation a key step in initiating the cytotoxic response against hematologic malignant cells, which often fail to effectively present self-antigens of the affected organism [126,144,149]. At the same time, cDC1s present antigens in the context of MHC class II, which leads to the activation of CD4^+^ T cells and provides helper signals necessary to maintain and amplify the immune response [135].

Mature cDC1s migrate to lymph nodes, where they present antigens to T cells, providing three complementary activation signals: (1) peptide presentation in the MHC complex, (2) co-stimulation through CD80/CD86 interactions with lymphocyte’s CD28, and (3) cytokine secretion, primarily interleukin 12 (IL-12), which supports Th1 lymphocytes differentiation and enhances the cytotoxic activity of T cells and NK cells [149,150]. Due to these properties, cDC1s constitute a key DC subtype initiating an effective cytotoxic response and are one of the main targets of modern anticancer immunotherapies [143].

The identity program of cDC1 is governed by an integrated transcriptional axis composed of PU.1, IRF8, and BATF3, which collectively define their unique capacity for cross-presentation of antigens and activation of cytotoxic CD8^+^ T lymphocytes. PU.1 initiates chromatin opening and activation of regulatory elements, and IRF8 induces genes involved in antigen processing and MHC class I presentation, while BATF3 consolidates the effector program through sustained expression of *XCR1*, *CLEC9A,* and *CD8α* [151,152]. The cooperation among these TFs establishes a self-reinforcing regulatory network that maintains the cDC1 phenotype. Notably, the enforced activation of the PU.1–IRF8–BATF3 axis in malignant cells can re-establish this program, driving their conversion into immunogenic cDC1-like cells capable of initiating cytotoxic T cell responses and suppressing tumor progression [45,153,154].

Cells of another subtype, cDC2, present antigens primarily in the context of MHC class II, activating CD4^+^ T cells and shaping the direction of Th1, Th2, or Th17 lymphocyte responses. They also support the proliferation and function of CD8^+^ cytotoxic lymphocytes, making them an essential element in coordinating the adaptive immune response [128]. In the context of leukemia, cDC2s participate in modulating the activity of bone marrow helper lymphocytes, influencing the local balance between effector immunity and tumor tolerance [127,135].

The third type, DC3s, combines the features of cDC1s and cDC2s. They demonstrate the ability to activate T and B lymphocytes, but their specific role is the induction of tissue-resident memory (Trm) lymphocytes, which may play a prognostic and therapeutic role in hematological malignancies [155]. In leukemias, DC3s may support the development of immunological memory in the myeloid niche, potentially enhancing the efficacy of the secondary response after remission or immunotherapeutic treatment [128,155].

Other, minor DC classes are monocyte-derived DC (moDC) and mature DC enriched in immunoregulatory molecules, mregDCs. Monocyte-derived DCs are found primarily in inflammatory conditions and in the tumor microenvironment, where they can partially assume antigen-presenting functions. However, in leukemias, they often exhibit immunoregulatory properties, promoting the development of Treg cells and supporting resistance to treatment, including radiotherapy [156]. In turn, mregDCs is a population characterized by the simultaneous expression of costimulatory and regulatory molecules. Their presence in the leukemic marrow microenvironment is associated with the formation of local immunosuppressive niches, favoring the maintenance of Tregs and limiting the effectiveness of immune surveillance. Modulating their function represents a promising therapeutic strategy aimed at reprogramming the DC phenotype from tolerogenic to immunostimulatory [127].

The bone marrow microenvironment significantly influences the activity and phenotype of dendritic cells. Under the influence of immunosuppressive cytokines such as IL-10 and TGF-β, DCs can adopt a tolerogenic phenotype that limits the activation of effector T cells and supports the development of regulatory T cells. Despite these limitations, DCs participate in maintaining long-term immunological memory; cDC2 and DC3 populations support the differentiation of T cells into memory cells and their reactivation upon disease relapse [128].

From a clinical perspective, dendritic cells represent a promising therapeutic target in leukemias. Strategies based on their stimulation include the administration of the growth factor agonists (aiming FLT3L, STING, and TLR) and cellular vaccines using DCs to present leukemic antigens. Furthermore, the ability to transdifferentiate monocytes or leukemic cells into cells with a cDC1-like phenotype may increase the efficiency of antigen presentation and enhance the activation of T cell responses, which translates into improved disease control [127,143].

## 6. Changing Cell Identity Toward a Dendritic Phenotype

### 6.1. Experimental Techniques

Desired, from the translational point of view, transdifferentiation of leukemic or somatic cell identity toward DCs is achieved using a combination of genetic, epigenetic, and environmental approaches that together induce direct reprogramming of transcriptional networks and epigenome remodeling.

**Figure 1:**
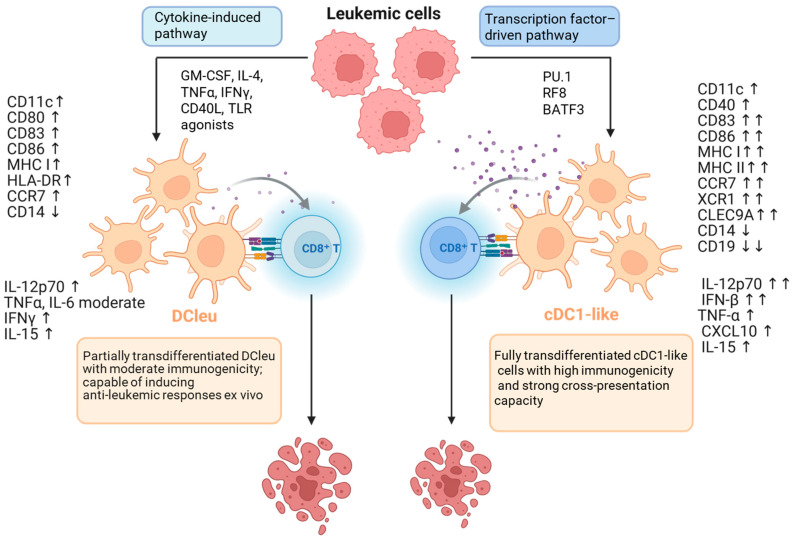
Two pathways of transdifferentiation of leukemia (AML) cells into dendritic cells (DCs). Left: the cytokine-driven (microenvironmental) pathway; stimulation with GM-CSF, IL-4 ± TNFα, CD40L, or TLR agonists activates the IRF8–BATF3 axis, leading to the generation of leukemia-derived dendritic cells (DCleu). These DCleu exhibit CD11c, CD86, and HLA-DR expression, secrete IL-12p70 and TNFα, and display moderate antigen-presenting capacity and T cell-stimulatory potential. This process reflects the intrinsic plasticity of leukemic cells under microenvironmental cues and enables their partial differentiation toward an immunogenic DC phenotype. Right: the transcription factor-driven pathway; enforced expression of C/EBPα, PU.1, IRF8, and BATF3 stably reprograms leukemic cells into cDC1-like cells characterized by expression of XCR1, CLEC9A, and CD141/BDCA3, along with strong secretion of IL-12 and IFN-β, enabling potent CD8+ T cell activation. Both mechanisms converge on the IRF8–BATF3 axis, but differ in the depth of epigenetic remodeling and stability of the immunogenic outcome. Analogous processes have been observed in B-ALL models, where the loss of PAX5 and activation of PU.1/IRF8 drive partial reprogramming of lymphoblasts into DC-like cells. Symbols at marker names reflect the direction and magnitude of change compared to the parental cell: moderate = moderate increase; ↑ = increase; ↑↑ = strong increase; ↓ = decrease; ↓↓ = strong decrease. (Based on [45,125,152,153,157,158,159].) Created in BioRender. Czogalla, A. (2025) https://BioRender.com/dwn4tyr, accessed on 11 December 2025.

The most commonly used method is to induce or enhance the expression of TFs that define dendritic cell identity. PU.1, IRF8, BATF3, and C/EBPα play a key role, activating programs typical of cDC1 and cDC2 [45,154] (see Figure 1). Experimental systems utilize lentiviral or adenoviral vectors to introduce genes encoding these TFs, enabling the conversion of fibroblasts, monocytes, or leukemic cells into cells with the morphology and function of DCs. Forced expression of *C/EBPα* and *PU.1* leads to the conversion of B lymphocytes into macrophages or DCs, a process accompanied by chromatin remodeling and a change in enhancer profile [48,125].

Direct reprogramming of leukemic cells requires breaking the “lineage memory”, which is why epigenetic modulators are used to lower the barrier to phenotypic switching. HDAC inhibitors (e.g., trichostatin A) and DNMT inhibitors (e.g., 5-azacytidine) increase chromatin accessibility and facilitate the activation of DC programs [160,161,162]. IDH1/2 inhibitors and TET modulators normalize α-KG/2-HG metabolites, restoring the ability to demethylate DNA at key immunogenic gene loci. Studies on AML have shown that combining demethylation with IRF8 expression can induce the expression of CD86, MHC II, and CCR7 markers typical of DCs [121].

The microenvironment plays a key role in the induction of DC identity. GM-CSF + IL-4, which activate the JAK–STAT pathway and promote the transition of monocytes into DCs, are most commonly used [127]. In cancer or leukemia settings, the addition of TNF-α, IL-6, IFN-γ, and TLR agonists (e.g., LPS or poly I:C) increases cell immunogenicity and supports the maturation of the DC phenotype. In AML and B-ALL, this approach led to the formation of the so-called leukemia-derived DCs (DCleu), capable of presenting tumor antigens and activating T lymphocytes [163,164].

The altered metabolic environment promotes cellular plasticity, therefore increasing NAD^+^ and acetyl-CoA through SIRT1 activators or mTOR modulation supporting DC maturation and stabilization of the antigen presentation program [165,166]. For example, in AML, induction of the oxidative pathway and reduction in glycolysis promote a transition to a DCleu phenotype with high MHC and CD80/CD86 expression [167].

The use of CRISPR/Cas9 allows for the selective silencing of identity gatekeepers (e.g., *PAX5, EBF1, IKZF1*) in B-derived cells, facilitating their transition toward either myeloid or dendritic cell lines. The combination of *PAX5* silencing and *C/EBPα* expression leads to a complete switch from the lymphoid to myeloid program [112]. Edits at the *IRF8* and *BATF3* loci enable the precise control of cDC1 or cDC2 conversion [154].

### 6.2. Transdifferentiation in the Therapies of Leukemias

From a clinical application perspective, the directionality of transdifferentiation has a dual significance. On the one hand, it poses a diagnostic challenge, leading to phenotypic instability, misclassification, and difficulty monitoring residual disease. On the other hand, it opens up therapeutic prospects because it enables targeted reprogramming of leukemic cells toward highly immunogenic states, promotes the fixation of target antigens, and limits mechanisms of immune evasion. Thus, transdifferentiation becomes the basis for the development of new immunotherapy strategies in leukemias [168,169,170].

The number and functionality of cDC1 in both peripheral blood and bone marrow microenvironment strongly correlate with patient prognosis and the efficacy of leukemia immunotherapy [171]. Clinical studies have shown that patients with acute myeloid leukemia (AML) with a higher percentage of cDC1 and higher expression of activation molecules such as CD86 and HLA-DR are more likely to respond to targeted immunotherapies, including PD-1 inhibitors and peptide vaccines, and achieve longer remissions [143,171]. Therapeutic strategies aimed at enhancing cDC1 function focus on increasing their number and activity in the tumor microenvironment. Administration of the growth factor FLT3L induces expansion of the cDC1 population, while activation of the TLR3, STING, or CD40 pathways enhances their ability to secrete IL-12 and promote Th1 differentiation [127,143,172]. Combined with targeted antigen delivery via DEC-205 or CLEC9A receptors, potent and sustained priming of CD8^+^ T cells can be achieved [127].

In parallel, increasing attention is being paid to modulating other dendritic cell subtypes, which often adopt an immunosuppressive phenotype in leukemic conditions. pDCs demonstrate reduced production of type I interferons, which limits their ability to activate effector cells. Their reactivation with TLR7/9 or STING agonists may restore IFN-I secretion and increase the efficacy of antitumor therapy [141].

In turn, moDCs and mregDCs show increased expression of inhibitory molecules such as PD-L1, ILT3, and IDO1, which promote the development of Tregs and suppress the cytotoxicity of CD8^+^ cells. Direct reprogramming of these cells toward a cDC1-like phenotype using CD40 agonists, GM-CSF, and IL-12 can significantly enhance their antigen-presenting capacity and enhance the antitumor response in combination therapy [143,156].

From a translational perspective, the presence of active cDC1 and the balance between immunostimulatory and regulatory DC subtypes are key determinants of immunotherapy success. Combining cDC1-stimulating strategies with effector therapies, such as CAR-T, BiTEs, or checkpoint inhibitors, creates a synergistic system that enhances leukemic clone recognition and increases the durability of the cytotoxic response [173]. From a clinical perspective, this means that modulating DC activity and differentiation, particularly toward cDC1-like phenotypes, represents a key direction for the development of modern immune therapies against leukemia. Integrating these approaches with effector therapy and MRD surveillance may enable not only deep but also durable remissions in the future.

The most commonly used approach is to generate autologous moDCs loaded with leukemic antigens, peptides, mRNA, or cell lysates [164,174]. The efficacy of these vaccines depends on the degree of DC maturation, evidenced by the expression of CD80, CD86, CD40, and CCR7, and the ability to secrete IL-12, crucial for effective priming of CD8^+^ lymphocytes [149].

An additional and particularly promising strategy involves the generation of DC–leukemia cell fusions. This approach merges the antigenic repertoire of leukemic cells with the potent presentation and costimulatory capacity of mature dendritic cells, minimizing epitope loss and enhancing T cell activation [175,176]. Such fusion vaccines effectively link tumor specificity with strong immunogenic potential, offering a means to overcome tolerance and elicit durable cytotoxic responses.

Other strategies combine antigen presentation with a danger signal—DCs exposed to apoptotic or necrotic leukemic cells mature faster and secrete IL-12, which enhances the Th1 response [146,149].

Alternatively, leukemia-derived DCs, i.e., leukemia cells differentiated in vitro toward a DC-like phenotype with GM-CSF, IL-4, TNF-α, and CD40L, are used. Although they provide full antigenic compatibility, their limited IL-12 production and migration capacity remain a challenge [164].

Of particular importance are cDC1-targeted strategies that ensure the most efficient antigen cross-presentation and coupling of the NK–cDC1–TCD8^+^ axis. They can be induced ex vivo by polarizing moDCs to a cDC1-like phenotype or in vivo by using FLT3L and TLR3, STING, and CD40 agonists with targeted antigen delivery via DEC-205 or CLEC9A [143]. Currently, the greatest effects are achieved by combination approaches, integrating the priming stage with effector interventions such as CAR-T, BiTEs, or checkpoint inhibitors, and with the phase of consolidating immunological memory [135].

Transdifferentiation-based immunotherapies are currently limited to ex vivo-based procedures, such as the construction of DC-based vaccines (see below). As shown in Figure 1, the main procedures involve cytokines or TFs. In some cases, low-molecular-weight substances such as methotrexate have been used to carry out this process [177]. However, the prospects for in vivo manipulation of cytokine or small-molecule levels are rather limited due to the very low specificity of such substances. The use of viral vectors, such as lentiviral vectors or, in particular, recombinant adeno-associated virus (AAV) vectors, seems more promising, as these vectors are characterized by high transduction efficiency. Recombinant AAV-based therapeutics are intended for single-dose gene therapy, as these viruses are harmless to humans and animals and have tropism for most cells and tissues [178]. Immune responses to AAV and the resulting transgene, i.e., innate, adaptive, and delayed, may preclude the use of such constructs. Moreover, many individuals possess neutralizing antibodies and/or memory cells that protect against natural AAV infections [179,180]. However, these vectors effectively deliver the transgene (in this case, TF sequences) to most tissues and organs, which causes a lack of specificity and, therefore, the “off-target effect.” This problem is being addressed by attempts to construct viral vectors, for example, those encoding RGD peptides [181], or target antibodies binding protein A [182].

Nonviral vectors include a wide variety of nanomolecular assemblies, such as organic and inorganic nanoparticles, dendrimers, and lipid nanoparticles. The main components of a later class of nanomolecular assemblies are liposomes and the so-called lipid nanoparticles (for a review, see [180]). Therapeutically useful liposomes are single vesicles of the lipid bilayer with a diameter < 100 nm, containing an aqueous space within. Long-circulating liposomes are obtained, including a high-molecular-weight (1–5 kDa) PEG lipid derivative. These assemblies can be derivatized to contain various targeting molecules, such as humanized monoclonal antibodies, e.g., [183,184], or various targeting molecules [185]. Liposomes, however, have relatively low transfection efficiency, which is why lipid nanoparticles were developed. Although these lipid and nucleic acid transport units are highly efficient in transfection and have been used in FDA-approved drugs, these nanoparticles are prone to liver accumulation [186]. Recently, several studies have been published proposing targetable, PEG-decorated lipid nanoparticles for gene transport (review, [187]). Such constructs seem promising for efficient, targeted transport of TF-encoding vectors.

### 6.3. Dendritic Cell-Based Immunotherapies

DC-based immunotherapies are among the most advanced and promising areas of modern immuno-oncology. Dendritic cells are key APCs responsible for the initiation and modulation of both innate and adaptive immune responses. Their ability to activate CD8^+^ and CD4^+^ T cells and coordinate the interaction between cellular and humoral immunity has led to their development as the basis for many modern therapeutic strategies in oncology [127,188,189,190].

The most comprehensive clinical demonstration of this concept is Sipuleucel-T (Provenge^®^), the first and only DC-based immunotherapy approved for cancer treatment. It was approved by the FDA in 2010 for the treatment of castration-resistant prostate cancer in asymptomatic or minimally symptomatic patients. Its mechanism of action involves the use of autologous APCs, primarily monocytes and DCs, activated ex vivo with recombinant prostatic acid phosphatase (PAP) antigen linked to GM-CSF. When reintroduced to the patient, the activated cells initiate a specific immune response directed against prostate cancer cells [191,192,193]. In clinical trials, Sipuleucel-T extended median overall survival by approximately 4.1 months compared to placebo, while maintaining a favorable safety profile [192]. Sipuleucel-T therapy is considered a milestone in the development of cancer immunotherapy because it was clinically proven for the first time that activating a patient’s immune system with DCs can translate into improved overall survival. The success of this approach has become a benchmark for the development of subsequent DC-based strategies in oncology, including therapies directed against hematological malignancies such as acute leukemia and multiple myeloma [127,143].

In the context of hematological diseases, no DC therapy has yet been clinically approved, but several strategies are in advanced stages of research. Among the best clinically documented are two therapies: DC/AML fusion vaccines, an autologous fusion of DC with leukemic cells, demonstrating safety, the ability to activate CD8^+^ T cells, and control MRD [194], and the other is DCP-001, an allogeneic AML-derived product, an “off-the-shelf” vaccine that combines safety with a broad antigenic profile [195,196,197].

In parallel, personalized strategies are being developed, such as WT1-DC and mRNA-DC, enabling precise targeting of leukemic antigens (WT1, PRAME, RHAMM) and combining them with adoptive therapies or checkpoint inhibitors [198,199,200,201]. In the case of multiple myeloma, the most advanced concept is DC/MM fusion vaccines, which are the only ones to have reached Phase II clinical trials, confirming their efficacy in activating the cytotoxic response and controlling MRD [202].

Currently, most DC vaccines are tested in the remission phase, following auto- or allo-HCT (hematopoietic cell transplant), or for the treatment of residual disease, where the bone marrow microenvironment favors an effective immune response. In the active phase of disease, efficacy is limited by severe immunosuppression. Therefore, DC therapies work best in combination regimens including PD-1/PD-L1 inhibitors, lenalidomide, or adoptive immunotherapies, which enhance the activity of the cDC1–CD8^+^–NK axis and increase the chances of achieving durable remission [127,143].

In this context, approaches such as DC/AML fusion vaccines or allogeneic platforms such as DCP-001 can be viewed as hematological equivalents of a concept already clinically validated by Sipuleucel-T [174,195]. More details are provided in the next paragraph.

Although Sipuleucel-T was developed and approved for the treatment of prostate cancer, its clinical success has important implications for the development of dendritic cell-based immunotherapies for leukemia [192,203]. Key elements of this approach, including the use of autologous cells, broad presentation of tumor antigens, and administration of therapy in the setting of low tumor burden and relatively preserved immune competence, provide direct inspiration for dendritic cell (DC)-based strategies currently under development for the treatment of leukemia [171,197,204].

In summary, although Sipuleucel-T remains the only approved DC therapy, it is pioneering the development of new immunotherapies and off-the-shelf strategies. This development is directed toward personalized and combination therapies that integrate the presentation properties of DCs with control of the immunosuppressive tumor microenvironment.

### 6.4. Clinical Trials in AML

In the context of hematological diseases, no DC therapy has yet been clinically approved, but several strategies are in advanced stages of research. Recently, several clinical trials have been conducted to evaluate the safety, immunogenicity, and potential clinical efficacy of DC-based vaccines in patients with AML. These studies have primarily focused on clinical situations characterized by low disease burden, including complete remission after chemotherapy, allogeneic hematopoietic stem cell transplantation (allo-HCT), and maintenance therapy for MRD control.

Among the best clinically documented is the DC/AML fusion vaccine, an autologous fusion of DCs with leukemia cells, demonstrating safety, the ability to activate CD8^+^ T cells, and control MRD [194]. Early phase clinical trials of DC/AML fusion vaccines have demonstrated favorable safety profiles and consistent induction of leukemia-specific CD4^+^ and CD8^+^ T cell responses. In several studies of AML patients in remission after chemotherapy, vaccination was associated with MRD control and evidence of prolonged remission duration compared with observation alone [174,204]. Importantly, DC/AML fusion vaccines have also been evaluated after allo-HCT, where they were generally well tolerated and associated with maintenance of remission in the majority of vaccinated patients, without clear evidence of increase severity symptoms of graft-versus-host disease (see also Table 1).

The other most thoroughly studied approach is DCP-001, an allogeneic AML-derived product and an “off-the-shelf” vaccine that combines safety with a broad antigenic profile. DCP-001 is cryopreserved, gamma-irradiated allogeneic DC derived from an AML cell line (DCOne) with broad antigen presentation. Phase I–II studies in elderly or high-risk AML patients in complete remission, including those with detectable MRD, have confirmed the good tolerability and immunogenicity of DCP-001. Importantly, MRD conversion and signals of improved relapse-free survival were observed in a subset of patients, justifying further clinical evaluation of this platform [195,196,197] (see also Table 1).

A summary of the most advanced clinical trials evaluating DC/AML fusion vaccines and DCP-001, including enrollment status, key efficacy endpoints, and safety profiles, is presented in Table 1.

The available clinical data indicate that dendritic cell-based vaccines in AML are safe and immunogenic, with the most pronounced clinical benefit observed after remission and in the setting of MRD. These findings support the further development of dendritic cell-based strategies, particularly in combination regimens aimed at overcoming immunosuppression and achieving durable disease control.

## 7. Conclusions and Perspectives

The abovementioned facts suggest a crucial role for transdifferentiation in the biology and pathology of progenitor cells and blood cells. Furthermore, transdifferentiation opens up a number of innovative therapeutic approaches. The most promising therapeutic axis involves lymphoid-to-myeloid/cDC1 conversion, driven by master regulators such as C/EBPα/β and the PU.1-IRF8-BATF3 transcriptional network. Research findings from both hematological and solid tumor models consistently demonstrate that enforcing the antigen presentation program in malignant cells can trigger effective immune activation and even induce long-term immunological memory. Importantly, this conversion can occur not only ex vivo but also in vivo. Consequently, leukemia reprogramming is emerging as a new paradigm in immunotherapy, where malignant cells are transformed into autologous immunostimulatory cells.

Leukemic cells/blasts exhibit a high degree of phenotypic plasticity, allowing them to adapt to changing microenvironmental conditions and evade immune surveillance, which is one of the main challenges in leukemia therapy. One of the key mechanisms underlying the plasticity/flexibility of leukemia is transdifferentiation, or direct reprogramming, a process in which cells change their phenotypic identity without returning to pluripotency. Transdifferentiation can be pathological, leading to a change in cell lineage (e.g., from lymphoid to myeloid), which promotes drug resistance and disease relapse. In turn, transdifferentiation, especially in the context of hematopoiesis, can serve as a therapeutic mechanism. Induction of reprogramming leukemic cells in vivo or in vitro toward immunogenic phenotypes, such as DCs, can enhance tumor antigen presentation and promote T and NK cell activation, resulting in immune-mediated elimination of tumor cells. Combining transdifferentiation-based approaches with advanced immunotherapies, including CAR-T cells, BiTE, or checkpoint inhibitors, represents a promising strategy for overcoming immune evasion in leukemia.

A major challenge is understanding the molecular basis of transdifferentiation, including the role of TFs (PU.1, C/EBPα, GATA2, RUNX1) and epigenetic regulators that enable cell lineage reprogramming, as well as the hematopoietic growth factors and cytokines. Deciphering these mechanisms could contribute to the development of new therapies that not only eliminate leukemic cells but also modify their phenotypes to make them more susceptible to immune system attacks. One of the important questions is whether it would be possible to reconstitute of entire cell membrane of transdifferentiated, leukemia-derived cells in the artificial membrane to stimulate innate immune response (CD8-T, NK, and other cells).

Several DC-based therapeutic strategies are currently in advanced development. These include DC/AML fusion vaccines, autologous DC fusions with leukemic cells, and DCP-001, an allogeneic AML-derived product. Moreover, personalized strategies, such as WT1-DC and mRNA-DC, are also available, and targeting leukemic antigens (WT1, PRAME, RHAMM) are available, also in combination with adoptive therapies or checkpoint inhibitors. These studies are pioneering near-term therapeutic successes for leukemias and other cancers.

In summary, transdifferentiation in leukemia represents a dual phenomenon: on the one hand, an adaptive mechanism driving malignant tumor survival and treatment resistance, and on the other hand, a potential avenue for innovative therapies. Mastering this process could define a new generation of immunotherapies that leverage the inherent plasticity of leukemic cells to achieve therapeutic benefit.

## Figures and Tables

**Table 1:** Clinical trials of dendritic cell-based vaccines in acute myeloid leukemia (AML). Efficacy outcomes are reported as signals and immunological correlates, where applicable. Toxicity grades refer to the Common Terminology Criteria for Adverse Events (CTCAE). Abbreviations: AML, acute myeloid leukemia; CR, complete remission; MRD, measurable residual disease; allo-HCT, allogeneic hematopoietic stem cell transplantation; GVHD, graft-versus-host disease; MDS, myelodysplastic syndrome.

Therapeutic Strategy (Clinical Trial ID)	Clinical Setting	Phase/Recruitment Status	Efficacy (Key Endpoints)	Safety	References
WT1 mRNA–electroporated dendritic cell vaccine (NCT00965224)	AML in complete remission after chemotherapy	Phase II; recruitment completed	Induction of WT1-specific CD4^+^ and CD8^+^ T cell responses; molecular and immunological remission in a subset of patients; signals of prolonged relapse-free survival in immunological responders	Predominantly grade 1–2 local and flu-like adverse events; no consistent vaccine-related ≥ grade 3 toxicities reported	[174,204]
DC/AML fusion vaccine post allo-HCT (NCT03679650)	AML after allogeneic hematopoietic stem cell transplantation	Phase I; recruitment completed (follow-up ongoing)	Induction of leukemia-specific T cell responses; the majority of vaccinated patients remained in remission during the observation period	Acceptable safety profile; mainly grade 1–2 adverse events (injection-site reactions); cases of acute and chronic GVHD reported, including events assessed as possibly related to vaccination	[205]
Allogeneic DC vaccine DCP-001 (NCT01373515)	AML in complete remission or at high risk of relapse	Phase I; recruitment completed	Broad immunogenicity; improvement in residual disease-related parameters in a subset of patients; clinical signals of prolonged relapse-free survival in patients with low disease burden	Well tolerated; predominantly grade 1–2 adverse events; no vaccine-related ≥ grade 3 toxicities reported	[197]
Allogeneic DC vaccine DCP-001—observational follow-up (NCT01373515)	High-risk AML/MDS	Observational long-term follow-up; completed	Durable immune responses; clinical signals of improved overall and relapse-free survival	No new safety signals identified during long-term follow-up	[195]
Allogeneic DC vaccine DCP-001—ADVANCE II)	AML in complete remission with measurable residual disease	Phase II/IIb; recruitment ongoing	Primary endpoints include relapse-free survival and MRD dynamics (analysis ongoing)	Safety evaluation ongoing; no unexpected safety signals reported to date	[206]

## Data Availability

No new data were created or analyzed in this study. Data sharing is not applicable to this article.
